# Prediction of Protein–Protein Interactions Between Alsin DH/PH and Rac1 and Resulting Protein Dynamics

**DOI:** 10.3389/fnmol.2021.772122

**Published:** 2022-01-20

**Authors:** Marco Cannariato, Marcello Miceli, Marco Cavaglià, Marco A. Deriu

**Affiliations:** PolitoBIOMed Laboratory, Department of Mechanical and Aerospace Engineering, Politecnico di Torino, Turin, Italy

**Keywords:** IAHSP, rare disease (RD), protein, molecular dynamics, DH/PH

## Abstract

Alsin is a protein of 1,657 amino acids known for its crucial role in vesicular trafficking in neurons thanks to its ability to interact with two guanosine triphosphatases, Rac1 and Rab5. Evidence suggests that Rac1 can bind Alsin central region, composed by a Dbl Homology (DH) domain followed by a Pleckstrin Homology (PH) domain, leading to Alsin relocalization. However, Alsin three-dimensional structure and its relationship with known biological functions of this protein are still unknown. In this work, a homology model of the Alsin DH/PH domain was developed and studied through molecular dynamics both in the presence and in the absence of its binding partner, Rac1. Due to different conformations of DH domain, the presence of Rac1 seems to stabilize an open state of the protein, while the absence of its binding partner results in closed conformations. Furthermore, Rac1 interaction was able to reduce the fluctuations in the second conserved region of DH motif, which may be involved in the formation of a homodimer. Moreover, the dynamics of DH/PH was described through a Markov State Model to study the pathways linking the open and closed states. In conclusion, this work provided an all-atom model for the DH/PH domain of Alsin protein; moreover, molecular dynamics investigations suggested underlying molecular mechanisms in the signal transduction between Rac1 and Alsin, providing the basis for a deeper understanding of the whole structure–function relationship for Alsin protein.

## Introduction

Alsin is a protein of 1,657 amino acids encoded by the amyotrophic lateral sclerosis type 2 (ALS2) gene. Its functions are crucial for neuronal homeostasis; indeed, Alsin mutations have been correlated with neurodegenerative disorders such as infantile-onset ascending hereditary spastic paralysis (IAHSP) (Orrell, 1993). The sequence alignment has depicted this protein as folded into four structured domains, namely, the regulator of chromosome condensation 1-like domain (RLD), the Dbl homology and Pleckstrin homology domain (DH/PH), the membrane occupation and recognition nexus (MORN) motifs, and the vacuolar protein sorting 9 (VPS9) domain (Orrell, 1993; Sato et al., 2018). The ability of Alsin to interact with two GTPases, Rac1 and Rab5, is at the basis of its crucial role in vesicular trafficking, especially in neurons. In particular, Rac1 binding with DH/PH domain triggers the relocalization from the cytoplasm to the membrane, where Alsin acts as a Rab5 guanine-nucleotide exchange factor (GEF) (Kunita et al., 2007; Sato et al., 2018). Several studies have been carried out to investigate the physiological functions of this protein and how its mutation or loss leads to different forms of HSP (Cai, 2005; Otomo et al., 2008; Hadano et al., 2010; Fink, 2013; Gautam et al., 2015; Blackstone, 2018; Hsu et al., 2018; Sato et al., 2018). At the same time, the knowledge of the molecular mechanisms underlying Alsin biological functions and the nanoscale effect of mutations is crucial to design potential therapeutic strategies. However, an experimental structure of this protein has not been developed yet. Before the release of the AlphaFold protein structure database (Jumper et al., 2021), RLD was the only domain that had been modeled (Soares et al., 2009; Sato et al., 2018), while none of the Alsin regions has been studied exploiting molecular dynamics (MD) tools. The interaction between Rac1 and DH/PH domain is the first event of the pathways leading to the formation of early endosomes through Rab5 activation (Kunita et al., 2007). Indeed, it has been shown that to be redistributed on membrane ruffles, Alsin must be in a tetrameric form and interact with Rac1 (Sato et al., 2018). Interestingly, the DH/PH motif is characteristic of a family of Rho GEF, but Alsin was demonstrated to be an effector rather than GEF (Kunita et al., 2007). Previously, the dynamics of homologous domains from other proteins has been investigated, showing that it consists essentially of a collective motion of PH domain and the last residues of DH domain (Raimondi et al., 2015; Felline et al., 2019). Given the fundamental role of this region in Alsin biological functions and its different role from one of the similar motifs, this study aims to exploit homology modeling tools to build an atomistic model of the Alsin DH/PH domain and characterize its dynamics, both alone and in the presence of Rac1. To strengthen the results, the employed experimental setup has been tailored and validated replicating previous findings on a known RhoGEF oncoprotein, the leukemia-associated RhoGEF (LARG) (Kourlas et al., 2000; Ong et al., 2009). Moreover, a crystallographic structure showing this protein bound to its ligand partner, RhoA, has been used to model Alsin interaction with Rac1 (Kristelly et al., 2004). First of all, the Alsin dynamic has been compared with one of the LARG to understand the molecular basis of their different biological functions. Then, the conformations of the Alsin DH/PH region were analyzed both in the presence and in the absence of Rac1 to characterize the effect of such interaction at the nanoscale level. Finally, the dynamic of free Alsin was described through a Markov State Model to study the main states in which it could be found and discover the kinetics relationships between them. The results will provide an overall description of Alsin DH/PH domain possible conformations, both bound with Rac1 and alone. Furthermore, the putative molecular mechanism underlying the signal transduction between Rac1 and Alsin will be reported.

## Materials and Methods

Alsin DH/PH domain structure was obtained through homology modeling since no experimental structure is available. The homology model was used to study the effect of the interaction with Rac1 on Alsin dynamics. To this purpose, two systems were simulated with classical MD: Alsin DH/PH domain (Alsin^UnBnd^) and the DH/PH-Rac1 molecular complex (Alsin^Bnd^).

### DH/PH and DH/PH-Rac1 Molecular Models

The amino acid sequence of human Alsin has been retrieved from UniProt database (id: Q96Q42), and the residues corresponding to the predicted DH/PH domain (aa 686–1,010) were extracted and numbered according to that of the whole Alsin. Then, the homology model was built giving the above-mentioned sequence as input to the I-Tasser suite (Zhang, 2008; Roy et al., 2010; Yang et al., 2013). Models have been evaluated by means of C-score and TM-Score according to previous literature (Zhang, 2008). Among the output models, the one with the highest C-score was retained. The secondary structure of the homology model was analyzed through the STRIDE software package (Frishman and Argos, 1995). The quality of the model was evaluated by the suite Molecular Operating Environment (MOE) through the visualization of the Ramachandran plot, which represents the distribution of phi and psi angles pairs and the allowed regions. The percentage of residues lying in not allowed regions were compared with those of the templates used by I-Tasser during the construction of the model. Finally, for each template, the identity and similarity scores relative to Alsin were computed using MOE with the following procedure: the crystal structures of the templates were retrieved from Protein Data Bank, their sequences were aligned with one of the Alsin DH/PH domain, the amino acids outside the region covered by Alsin residues were deleted, and then the scores were computed by dividing the length of Alsin sequence. BLOSUM-62 score matrix was used to perform the alignment and compute the similarity scores (Henikoff and Henikoff, 1992). MOE software (ULC, 2019) was employed for model refining and molecular system setup.

**FIGURE 1:**
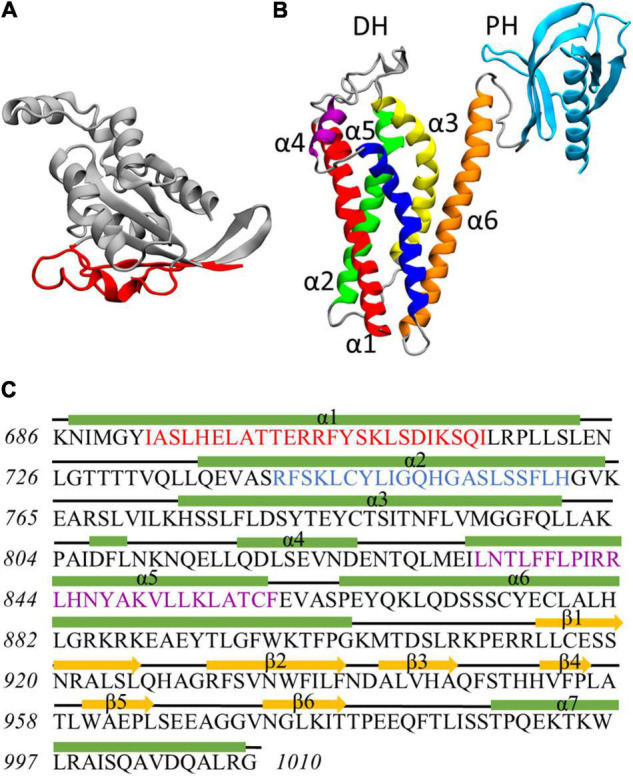
**(A)** Rac1 structure at the end of the dynamics. Residues exposed at the DH/PH-interacting surface are highlighted in red. **(B)** Homology model of Alsin DH/PH domain. The helices α1, α2, α3, α4, α5, and α6 of DH domain are colored in red, green, yellow, purple, blue, and orange, respectively. PH domain is colored in cyan. **(C)** Definition of secondary structure for Alsin DH/PH domain. Helices are represented by green rectangles, coils are represented by black lines, and strands are represented by yellow arrows. The conserved regions CR1, CR2, and CR3 are highlighted in red, blue, and purple, respectively.

Alsin^UnBnd^ initial configuration was obtained by adjusting the protonation state of the homology model according to a physiological pH of 7.4. The atomic coordinates of human Rac1 were obtained from Protein Data Bank (PDB: 3TH5, chain A; Krauthammer et al., 2012), and then, the nucleotide and magnesium ion were removed to study the interaction with the nucleotide-free GTPase as done in previous literature (Felline et al., 2019; Figure 1A). This crystallographic structure was previously employed to study Rac1 interaction with a binding partner through MD simulations (Ozdemir et al., 2018). To obtain the initial configuration of Alsin^Bnd^, the DH/PH domain homology model and Rac1 were superimposed (rigid-roto-translation) to LARG and RhoA (PDB: 1X86, chains A and B; Kristelly et al., 2004), respectively, as proposed previously (Gheyouche et al., 2021). Then, the protonation state was adjusted and, to avoid steric clashes due to superimposition, the potential energy was minimized.

### System Setup and Molecular Dynamics

Atomic positions for both systems, Alsin^UnBnd^ and Alsin^Bnd^, were retrieved from the output of molecular models building procedure. Topology was built by employing the AMBER ff99SB-ILDN force field (Lindorff-Larsen et al., 2010). The protein was inserted in a cubic box with periodic boundary conditions defined setting a minimum distance of 1 nm between the protein and the box edge. Then, it was solvated in explicit TIP3P water (Jorgensen et al., 1983), and subsequently, an appropriate number of Na^+^ and Cl^–^ were added to reach a physiological concentration of 0.15 M and to neutralize the charge. The energy minimization was performed through the steepest descend method for 2,000 steps. Then, two replicas were obtained as follows. An initial simulation of 500 ps in NVT ensemble and a following one of 500 ps in NPT ensemble were carried out, both of them under position restraints of alpha carbon. The NVT simulation was performed at a reference temperature of 300 K (τ = 0.1 ps) using the modified Berendsen thermostat (Berendsen et al., 1984). The NPT simulation was carried out at 1.0 bar using the Berendsen barostat with isotropic coupling (τ = 1.0 ps). Finally, an MD simulation in NPT ensemble was produced for 500 ns. The equation of motion was integrated with the leapfrog algorithm using a time step of 2 fs. Electrostatic interactions were treated with particle mesh Ewald method, short-range cutoff at 1.2 nm, and a switching of the potential starting at 1.0 nm. Van der Waals interactions were treated with a cutoff at 1.2 nm and a switching of the potential starting at 1.0 nm. The simulation engine employed was GROMACS 2020.4 (Lindahl et al., 2020).

For each system, two 500 ns long replicas were produced. To better explore the state space in Alsin^UnBnd^ system, four additional MD simulations, each one 100 ns long, were performed using initial configurations extracted from the equilibrium trajectories of Alsin^UnBnd^ replicas. The two 500 ns long trajectories will be called “long replicas,” while the four 100 ns long trajectories will be called “short replicas.”

DH/PH-Rac1 protein dynamics results, obtained in this work, have been compared with previous computational investigations on similar molecular complexes such as the DH/PH domain of the LARG (Felline et al., 2019) (comparison of results on LARG dynamics with previous literature is extensively reported in Supplementary Material SM1).

### Simulation Analysis

Visual molecular dynamics (VMD) (Humphrey et al., 1996) was used for a qualitative inspection of molecular systems and related trajectories.

For each investigated system (Alsin^Bnd^ and Alsin^Unbnd^), the structural stability throughout the MD simulation was evaluated through the root-mean-square deviation (RMSD) from the initial configuration of C-alphas atomic positions during the trajectory. Since previously it has been observed that the essential dynamics of the DH/PH domain that are expressed in other proteins is characterized by a collective motion of PH domain and α6 helix (Figure 1B), the RMSD was computed also to the C-alphas of the sole DH domain (residues 686–895). From the visual inspection of RMSD plots (Supplementary Figure SM2 1), the last 450 ns of each long trajectory was considered in the following analysis. For short trajectories, the last 90 ns were used in the analysis. The root-mean-square fluctuation (RMSF) and the force constants were computed as done in previous work (Felline et al., 2019) for each long replica, and then, the results were averaged (Supplementary Material SM2 Section 2). The probability of Alsin residues to be in contact with Rac1 was computed by sampling the MD trajectory every 250 ps as already done in previous literature (Deriu et al., 2014). For each sample snapshot, the distances between the atoms of one Alsin residue and the atoms of Rac1 were computed: the residue was in contact if at least one of the residue–residue distances was lower than a threshold of 0.3 nm (Deriu et al., 2014). The number of snapshots in which a residue was in contact divided by the total number of snapshots was the contact probability for that residue.

The position of PH domain with respect to DH region in the bound and unbound states has been investigated to understand the effect of Rac1 in the conformations of Alsin DH/PH domain. In the analysis of PH–DH relative position, the short replicas were considered in order to obtain a wider sampling of the state space. The potential mean force (PMF) of Alsin^UnBnd^ and Alsin^Bnd^ along an angular coordinate named α_*xy*_ and a relative position named PH_z_ (see Section “Effect of Rac1 interaction on PH dynamics” for an extensive definition of the coordinates and their visual representation) has been computed through Boltzmann inversion as:


(1) $$\text{PMF}\,\left(\alpha_{x\,y},{\text{PH}}_{z}\right)=-k_{B}\,T\,\ln p\,\left(\alpha_{x\,y},{\text{PH}}_{z}\right)$$

where *k*_*B*_ is the Boltzmann’s constant, *T* is the temperature, and *p*(α_*xy*_, PH_*z*_) is the probability distribution obtained from the histogram of MD data, where bins of 1° and 0.1 nm were used to discretize the state space along α_*xy*_ and PH_*z*_ directions, respectively.

As mentioned before, helix α6 motion characterizes the essential dynamics of homologous domains. Moreover, interesting regions were helix α3 and the following coiled region (α3-5) due to their observed dynamics and interaction with Rac1. Therefore, to evaluate the effect of the interaction between Rac1 and helix α6 (residues 865–895) on its straightness, the curvature of α6 axis on plane *xz* was analyzed as done previously (Ribarics et al., 2015) (Supplementary Material SM2 Section 3). Then, the effect of Rac1 interaction on α3 and α3-5 positions was described computing two quantities from representative snapshots extracted every 50 ps for both long and short replicas. The first one is the relative position along *z* axis between DH domain center of mass, without considering the helix α6 as in the previous analysis, and the region of helix α3 in contact with Rac1 (residues 788–793), such that positive values indicate α3 being over DH center of mass. The second one is the distance between the centers of mass of α3-5 (residues 796–816) and PH domain.

To better understand the conformational transitions characterizing the domain, Alsin^UnBnd^ dynamics was investigated through a Markov State Model (MSM), describing the state space in terms of DH–PH relative position through the previous mentioned coordinates, which were computed every 10 ps for both long and short replicas (Zhang et al., 2019). *K*-centers algorithm was used to discretize the state space, sliding window method was used to compute the count matrix, and maximum-likelihood estimation was used to obtain the transition matrix. The optimal lag time was chosen for analyzing the largest implied timescale at lag times between 0.5 and 17.5 ns. Microstates were grouped using the Robust Perron Cluster Cluster Analysis (PCCA+) algorithm (Noé et al., 2007) choosing the number of states from the distribution of the slowest ten implied timescales at the optimal lag time. A new MSM was then estimated and validated through a Chapman-Kolmogorov test according to the literature (Metzner et al., 2009; Prinz et al., 2011; Wang et al., 2018) (Supplementary Material SM2 Section 4). Finally, transition path theory was applied to the validated model to identify the most probable transition pathways characterizing free Alsin DH/PH domain. Once the initial and final states of interest were chosen, the net flux matrix was computed. Then, it was used to find the most probable pathway from the starting to the final state through the Dijkstra algorithm.

GROMACS built-in tools were used to compute RMSD and RMSF. Contact probabilities, force constants, the coordinates defined to describe the system, and PMF were obtained using python libraries and custom-made scripts (Michaud-Agrawal et al., 2011; Gowers et al., 2016). MSMBuilder libraries were used to build the MSM and perform transition path theory (Beauchamp et al., 2011).

#### Plots and Figures

Three-dimensional representations of the proteins were rendered in VMD. Ramachandran plots were generated in MOE, while all other data plots were generated using Matplotlib library (Hunter, 2007). The network representation of the transition matrix was obtained through PyEMMA libraries (Scherer et al., 2015).

## Results

### Homology Model of Alsin DH/PH Domain

The homology model of Alsin DH/PH domain was built by I-Tasser using 16 templates (coordinates reported as Supplementary Material SM3). The best model was characterized by a C-score of 0.66, an estimated TM-score of 0.80 ± 0.09, and an estimated RMSD of 5.0 ± 3.2 Å. On average, the sequence identity and similarity between the templates and Alsin were 12.5 and 28.2%, respectively (Supplementary Table SM2 1). The quality of the structure was also investigated by observing its Ramachandran plot (Supplementary Figure SM2 3), computing the percentage of residues lying in not-allowed regions, and comparing this value with the average on the templates. The percentage of Ramachandran outliers in Alsin homology model and the average on the chosen templates were 1.5 and 0.8%, respectively. Therefore, from the analysis of the torsional angles, the quality of Alsin model is in line with the crystallographic structures of the templates. As in other proteins, the DH domain of Alsin is characterized by six α-helices (α1–α6) organized in an oblong bundle. While helices α1 and α5 are exposed on the same side of the domain, probably forming the Rac1-binding region as in other proteins, helix α2 is exposed in the opposite surface, which is involved in dimerization of other DH domains and, most likely, Alsin itself. The third helix of DH region exposes its N-terminus and C-terminus at the dimerization and Rac1-binding surfaces, respectively. Finally, the helix α6 is located on one side of the domain and is connected by a random coil region to the PH domain. The latter is composed by six antiparallel β-strands followed by one α-helix and is organized in a globular structure (Figure 1B). Therefore, the structure of Alsin DH/PH domain is characterized by the same motifs of other Rho GEF proteins. To identify the conserved regions in Alsin DH/PH domain, its amino acid sequence was aligned in MOE with BLOSUM-62 score matrix with the ones of the 16 templates used to build the model. Then, the residues forming the conserved regions of TIAM1 (Worthylake et al., 2000) were used to locate them in Alsin (Supplementary Figure SM2 4). CR1 and CR3 were characterized by a higher number of conserved residues among the analyzed proteins, while no amino acid was totally conserved in CR2. The secondary structure of the model, analyzed through STRIDE software package; the residues composing helices and strands; and the conserved regions are shown in Figure 1C.

### Rac1 Interaction and Mechanical Properties

**FIGURE 2:**
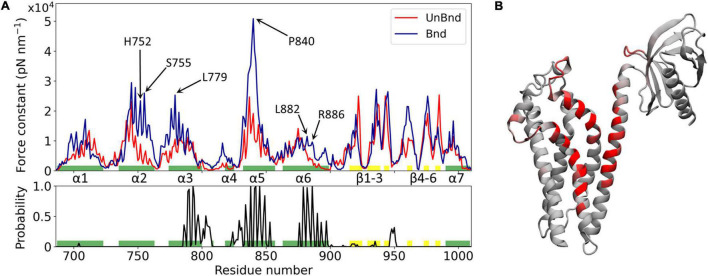
Effect of Rac1 interaction on Alsin DH/PH domain mechanical properties. **(A)** Alsin regions involved in the interaction with Rac1 are showed by the plot of the contact probability (down), while the force constant profiles of Alsin^UnBnd^ and Alsin^Bnd^ are compared (top). Residues forming helices and strands are colored in green and yellow, respectively. Peaks of force constant are highlighted for residues in contact with Rac1 or located in the putative dimerization surface. **(B)** Graphical representation of the main residues involved in the interaction with Rac1. Each residue is colored according to the contact probability, from 0 (gray) to 1 (red).

The initial configuration of Alsin^Bnd^ was modeled using the crystallographic structure of RhoA-bound LARG (Supplementary Figure SM2 5). The RMSD at the end of the superimposition were 1.98 Å between the DH/PH domains and 2.36 Å between the GTPases. The regions involved in the interaction of Alsin with Rac1 have been investigated computing the probability of each residue to be in contact with the GTPase (Figure 2). The amino acids with a contact probability greater than 0.9 were located in helices α3, α5, and α6 indicating that these are the main structures forming the Rac1-binding surface. Moreover, probabilities around 0.5 were found for three loops, the one immediately from C-terminal to helix α3, the one between helices α4 and α5, and, in PH domain, the portion from C-terminal to strand β3. Notably, helix α1 was not involved in significant interactions with Rac1 unlike LARG despite the starting configuration of Alsin^Bnd^ system was obtained from the crystallographic structure of RhoA-bound LARG (Supplementary Material SM1). Thus, between the conserved regions that are responsible for the interaction with Rho GTPase, only Alsin CR3 interacted with Rac1.

The mechanical properties at the single residue level of Alsin^Bnd^ and Alsin^UnBnd^ were inferred computing the force constants and then were compared to analyze the effect of Rac1 interaction on Alsin. Consistent with similar domains, the highest values were located in the structured regions, the presence of Rac1 increases on average the rigidity of the domain, and the greatest force constants were obtained in the first part of helix α5, independent of the GTPase presence (Figure 2). The most evident effect of Rac1 interaction was the increased mechanical rigidity within the first half of helix α5. In contrast, it was possible to observe only minimal changes in the mechanical profile of the first helix. Despite producing a contained increase in the force constants within its first half, the presence of Rac1 slightly increased the fluctuations of the following residues. Moreover, the second half of helix α6 and the region around helix α4 were characterized by higher force constants in the presence of Rac1. Limited differences could be observed within the PH domain, where in the absence of Rac1 the rigidity of strand β4 is lower while that of strand β6 is higher. Finally, in the bound state the mechanical properties within helix α2 and the first part of α3 were increased even though these regions did not interact with Rac1. Therefore, in the presence of Rac1, Alsin seems to increase the mechanical rigidity of residues exposed at the putative surface of dimerization. In particular, high mechanical rigidity residues were identified for the bound and unbound states as the ones with force constants greater than the average of the profile, as done previously (Felline et al., 2019). Then, these sets of residues were compared to find those identified in the Alsin^Bnd^ and not in Alsin^UnBnd^. The list of obtained amino acids with increased force constant, together with those characterized by high contact probability and conservation across the employed templates, is reported in Supplementary Table SM2 2. Interestingly, more than half of residues characterized by increased force constant are located in the CRs (68%), while only two highly conserved amino acids in helix α6, L882 and R886, are in contact with Rac1 and increased their mechanical rigidity due to this interaction.

The regions that characterize Alsin dynamics have been investigated through RMSF computed on C-alphas (Supplementary Figure SM2 6). The greatest fluctuations were located within PH domain both in the presence and in the absence of its binding partner, as in homologous domains (Felline et al., 2019). However, in the presence of Rac1, the flexibility of helix α6 was reduced indicating that the last residues of DH domain were less involved in the collective motion of PH region. In contrast, the coiled coil linker between the two domains contributed more to the dynamics in Alsin^Bnd^ than in Alsin^UnBnd^. Within DH domain, the region between helices α3 and α5 (α3-5) is characterized by higher fluctuations, reduced by the presence of Rac1. Finally, in the bound state, the fluctuations between β3 and β4 were reduced with respect to the unbound state.

### Effect of Rac1 Interaction on PH Dynamics

**FIGURE 3:**
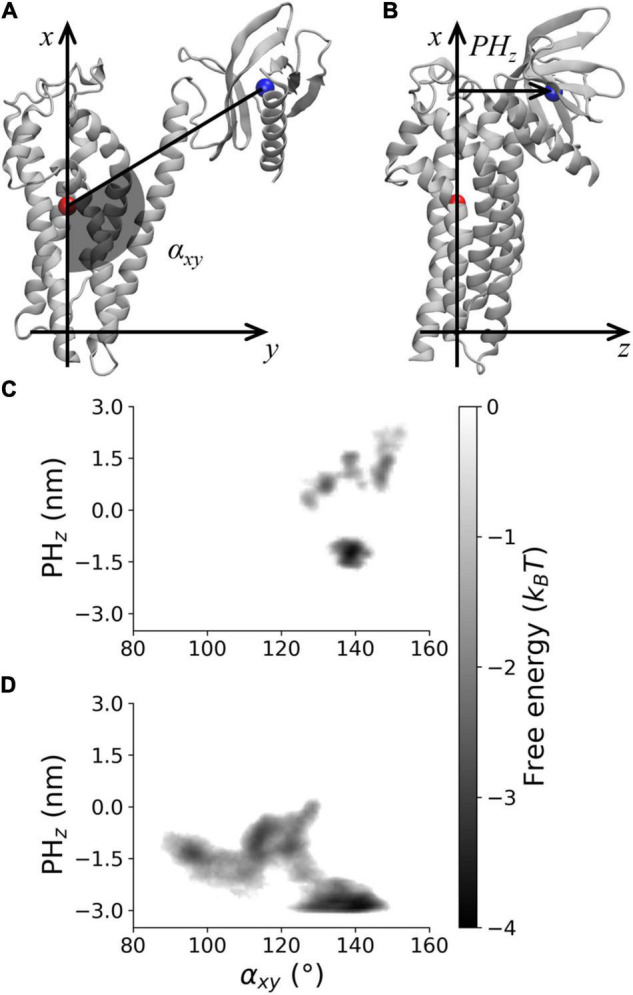
Visual rendering highlighting **(A)** α_*xy*_ and **(B)** *PH*_*z*_ and PMF for **(C)** Alsin^Bnd^ and **(D)** Alsin^UnBnd^.

The PMF along α_*xy*_ and PH_*z*_ was analyzed to characterize the DH–PH relative position in Alsin^UnBnd^ and Alsin^Bnd^ (Figure 3). To this purpose, the two coordinates were defined using a DH-based reference system. Identifying as *x* and *y* axis the first and second principal directions of the DH domain, the *z* axis is the one perpendicular to plane *xy*. α_*xy*_ has been defined as the angle in plane *xy* between the straight line parallel to the *x* axis passing through DH domain (residue 686–864) center of mass and the segment linking the latter to PH domain (residues 914–1,010) center of mass. Since the motion of last helix in DH region is involved in PH dynamics in other proteins, it was not considered when computing the center of mass to avoid possible changes in its position due to PH fluctuations (Figure 3A). Then, the coordinate PH_*z*_ was defined as the position along *z* axis of PH center of mass relative to DH center of mass, such that if this coordinate is positive, the PH domain is above the DH region center of mass and therefore closer to Rac1-binding surface (Figure 3B). It was possible to observe that only in the absence of Rac1, angles lower than 120° or PH_z_ lower than −2 nm was accessible. In contrast, positive PH_z_ was obtained almost only in Alsin^Bnd^ with the only exception of the region (α_*xy*_≈125°;PH_z_≈0). Notably, this is the only region of the energy profile in common between the two systems. The overall ability to explore different DH-PH relative positions is higher in Alsin^UnBnd^, where the minima are well connected. Instead, in the presence of Rac1, the minima are narrower and two low connected regions, characterized by positive and negative PH_z_, are explored. According to the definition of the two coordinates, the PMF showed that, in the absence of Rac1, more closed conformations were explored by the domain, i.e., the positions assumed by PH domain tended to move Alsin C-terminus closer to the N-terminus. On the other side, the presence of Rac1 seemed to stabilize a more linear and open conformation of the domain.

**FIGURE 4:**
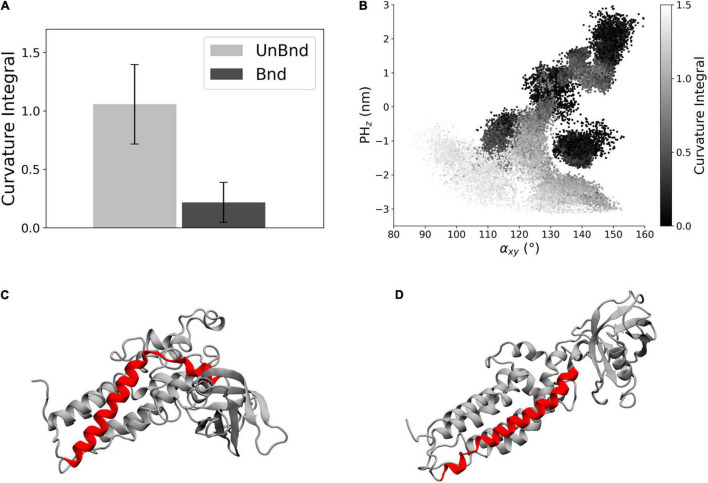
Analysis of helix α6 curvature. **(A)** Bar diagram showing the average curvature in the two states, where the error bars represent the standard deviation of the distribution. **(B)** Representation of the curvature integral value depending on the position of the protein in the α_*xy*_−*PH*_*z*_ plane. Each point is a snapshot of the trajectories and is colored according to the level of curvature of helix α6. **(C)** Representative snapshot of Alsin^UnBnd^ where α6 is highlighted in red. **(D)** Representative snapshot of Alsin^Bnd^ where α6 is highlighted in red.

The effect of the interaction between Rac1 and helix α6 has been investigated through the analysis of the helix axis curvature. The average curvature integral was significantly higher in Alsin^UnBnd^ (1.06 ± 0.34) than in Alsin^Bnd^ (0.22 ± 0.17) meaning that the presence of Rac1 stabilized a straighter conformation of α6 (Figure 4A). Moreover, the curvature integral in the UnBnd state tended to be higher in those conformations characterized by lower values of α_*xy*_ or PH_z_, i.e., within regions in which the domain is in a closed state (Figure 4B). At the same time, low values of curvature integral for Alsin^UnBnd^ were obtained in two regions, the one partially overlapping with Alsin^Bnd^ and the one characterized by α_*xy*_ ∈ [115,120] and PH_z_ ∈ [−1, 0]. Notably, the deepest minimum of Alsin^UnBnd^, in which the domain is closed, was characterized by some of the highest values of curvature integral, while the one of Alsin^Bnd^ by values approximately null. Therefore, through its action on the last helix of DH domain (Figures 4C,D), Rac1 was able to alter the dynamics of PH domain.

**FIGURE 5:**
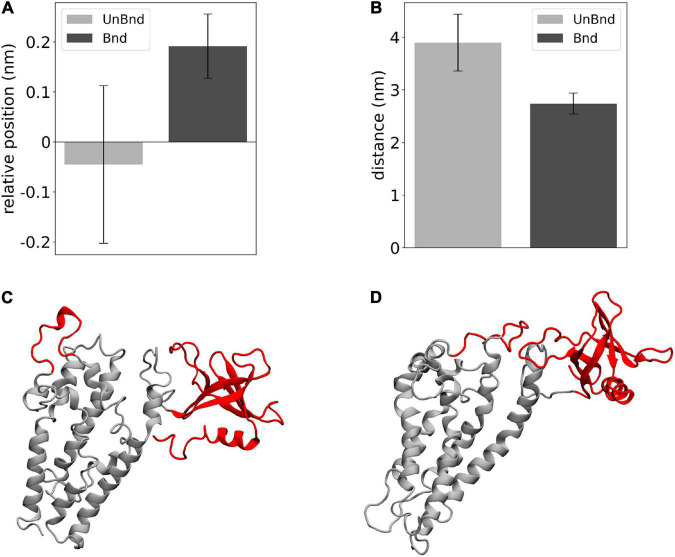
Analysis of the effect of Rac1-α**3** interaction. **(A)** Bar diagram showing the average DH-α3 relative position, where the error bars represent the standard deviation of the distribution. **(B)** Bar diagram showing the average PH-α3-5 distances, where the error bars represent the standard deviation of the distribution. **(C)** Representative snapshot of Alsin^UnBnd^ where α3-5 and PH are highlighted in red. **(D)** Representative snapshot of Alsin^Bnd^ where α3-5 and PH are highlighted in red.

Finally, the effect of Rac1 in the position of helix α3 has been described in terms of relative position along the *z* axis between its residues that were in contact with Rac1 and DH domain center of mass (Figure 5A). The average value was negative in Alsin^UnBnd^ (−0.05 ± 0.16 nm), while it was positive in Alsin^Bnd^ (0.19 ± 0.06 nm). The result of such Rac1-induced displacement has been described through the distance between α3-5 and PH domain (Figure 5B). It was possible to observe a difference between the two states, with lower values in the presence of Rac1 (2.74 ± 0.20 nm) than in case of free Alsin (3.90 ± 0.54 nm). Thus, the interaction with Rac1 stabilized the position of helix α3 above DH domain center of mass, and as a result, the region α3-5 moved closer to PH domain (Figures 5C,D).

### Markov State Model of Free Alsin

**FIGURE 6:**
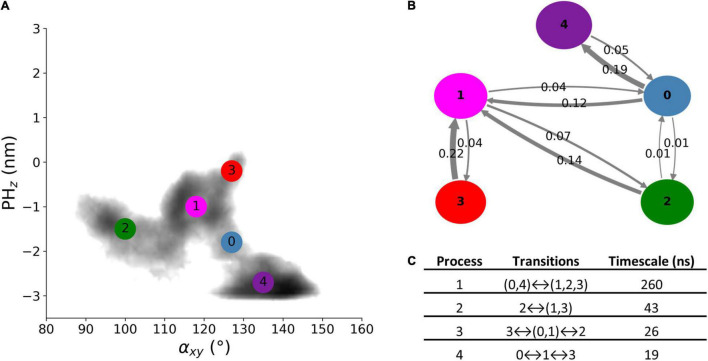
Alsin^UnBnd^ states from MSM analysis. **(A)** Location of the five states on the α_*xy*_−*PH*_*z*_ plane. The colors represent the free energy as in Figure 3. **(B)** Graphical representation of the transition matrix, where transition probabilities were rounded at the second decimal and probabilities lower than 0.005 were not considered. The arrow labels are the jump probabilities between states, the dimension of each sphere is proportional to the self-transition probabilities, and the arrow width is proportional to the probability to observe a jump between the states. **(C)** Timescales and transitions that characterize the four dynamical processes between the obtained states.

The dynamics of free Alsin was characterized through a MSM built describing the state space in terms of α_*xy*_ and PH_z_. From the analysis of the largest implied timescales at increasing lag times, the lag time to build the MSM was set to 9 ns. The number of states determined through PCCA+ algorithm was set to five according to the values of the first 10 implied timescales at 9 ns. The same lag time was used to build the MSM from the 5-state discretization (Supplementary Figure SM2 7). The model was validated through the Chapman-Kolmogorov test (Supplementary Figure SM2 8). State 3 corresponded to the region in which the free energy profile of Alsin^UnBnd^ and Alsin^Bnd^ were partially overlapped. It was characterized by a high probability to jump in state 1, while transitions from and to state 2 are less probable. State 1 was quite stable and communicated with all other states, with higher probabilities of jumping to state 2 or 0. In the former, the protein was closed on the side of DH domain, while the latter was the least stable state with a high transition probability toward state 4. The last state was quite stable and characterized by the protein being closed from the bottom of DH domain (Figures 6A,B). From the analysis of the right eigenvectors, it was possible to identify the four dynamical processes between the states. The slowest one, with an implied timescale of 260 ns, was the transition between states (0, 4) and (1, 2, 3). The second slowest process described the transition between states 2 and (1, 3) and had an implied timescale of 43 ns. The third one was the jump process between states (0, 1), 2, and 3, while the fastest process characterized the transition between states 0, 1, and 3. The latter were associates with 26 and 19 ns as timescales, respectively. A summary of the description of the four dynamical processes is reported in Figure 6C. Therefore, protein closure hiding the putative dimerization surface was the slowest process, while closure by the side of DH domain was faster. Transition path theory was applied to discover the most probable pathway associated with the transition between the most open conformations, represented by state 3, and the more closed ones, represented by states 2 and 4. The pathway connecting states 3 and 2 was 3→1→2, while the one connecting states 3 and 4 was 3→1→0→4. States 0 and 1 were also present in the most probable pathway from 2 to 4 and vice versa (Supplementary Figure SM2 9).

## Discussion

The availability of 3D atomistic models of Alsin is crucial to understand the molecular mechanisms at the basis of its biological functions. Indeed, the first step toward the treatment of ALS2-related pathologies such as IAHSP is a proper comprehension of the protein physiological behavior. In this work, we focused on Alsin DH/PH domain and developed its all-atom model through I-Tasser suite using 16 templates from RCSB database. The quality of the model was confirmed both by the confidence scores predicted by the employed software, especially the TM-score, and the analysis of the Ramachandran plot (Supplementary Figure SM2 3). The alignment of Alsin amino acid sequence with the templates (Supplementary Figure SM2 4) allowed us to locate the three conserved regions within the first, second, and fifth helices, in agreement with DH/PH domains of other proteins (Aghazadeh et al., 1998; Worthylake et al., 2000; Zhu et al., 2000; Kejin et al., 2001). It is worth mentioning that the performed alignment highlighted a highly conserved histidine (Alsin H752 in CR2), which might play a role in Alsin DH/PH-mediated self-oligomerization as already suggested by previous studies for the DH domain forefather, i.e., the proto-oncogene Dbl (Kejin et al., 2001).

Moreover, the present DH/PH model is in close agreement with the DH/PH domain of the Alsin model^1^ recently released on the AlphaFold protein structure database (Jumper et al., 2021). In a greater detail, the RMSD characterizing model superimposition is about:1.2 nm; 0.8 nm, and 0.6 nm for DH/PH, single DH, and single PH domain, respectively.

Since the interaction between Alsin DH domain and Rac1 is known to trigger relocalization at membrane level and activation of Rab5 through the C-terminal VPS9 domain (Sato et al., 2018), the effect of such interaction on the dynamics of Alsin DH/PH domain has been studied. The main regions involved in Alsin-Rac1 interactions were helices α3, α5, and α6; however, Rac1 bound also to non-structured regions even though they may have only a secondary role (Figure 2). Previous analysis on LARG was exploited not only to increase the consistency of our results but also as a term of comparison. Indeed, the first conserved region was in contact with the GTPase in the simulations of LARG (Supplementary Material SM1), but not in case of Alsin^Bnd^. Since CR1 has been described as crucial for the transforming activity of Rho GEF family of proteins (Zhu et al., 2000), these results may explain Alsin being a Rac1 effector. One effect of such interaction was the increased rigidity of the protein within not only two of the regions in contact with Rac1, α5 and α6, but also α2 and the first half of α3 (Figure 2). The latter are exposed on the surface opposite to the one interacting with Rac1, which was found to be involved in the dimerization of other DH domains (Kejin et al., 2001). Therefore, the mechanical properties of Alsin were locally altered by the interaction with Rac1, stabilizing the putative site of dimerization. Finally, fluctuations in the last residues of helix α6 are reduced, suggesting their involvement in the Rac1-driven conformational dynamics (Supplementary Figure SM2 6).

Previously, it has been proved that Alsin is sequestered in the cytoplasm due to an interaction between RLD and its C-terminus (Kunita et al., 2007). Rac1 signaling has been demonstrated to be fundamental for the recruitment of the tetrameric protein on membrane ruffles (Sato et al., 2018). Therefore, the role of Rac1-driven conformational dynamics of DH/PH domain in this signal transduction process has been investigated. The presence of Rac1 stabilized an open and linear conformation where, at most, the PH domain could slightly move above DH center of mass, near to Rac1 (Figure 3). In the absence of Rac1, different DH–PH relative positions were found and a wider area of the state space was explored. It is worth mentioning that, although the focus of the present work is Alsin conformational dynamics, the analysis of Rac1 dynamics and how it might be affected by the interaction with Alsin DH/PH is an interesting topic that should be addressed in the future.

The dynamics of Alsin^UnBnd^ was modeled through an MSM to describe the transitions between its different conformations. One open state was found in the region where the PMF of Alsin^Bnd^ and Alsin^UnBnd^ partially overlapped, while two closed states were detected (Figure 6). In one of them, the N- and C-terminals of the protein might interact by the side of the DH domain, while in the second, they might interact by the bottom of the domain. Notably, the latter corresponded with the deepest minimum of the free energy profile. Therefore, in the most stable conformation of Alsin^UnBnd^, the PH domain tends to move closer to a region that is involved in the homodimer formation in homologous proteins. In contrast, the Rac1-induced conformational change seems to stabilize the DH/PH domain toward a linear conformation. The closed and open conformations were connected mainly by one intermediate state, which allowed transitions both between the two closed states and from the open to the closed ones (Supplementary Figure SM2 9).

In particular, two main regions within the DH domain were characterized by different conformations in Alsin^UnBnd^ and Alsin^Bnd^. One of them was helix α6, i.e., the one linked to PH domain, whose curvature was higher in the absence of Rac1 and, particularly, in the closed states (Figures 4A,B). Since this helix was involved in the interaction with Rac1, it is possible that its bending is correlated with not only PH motion but also the propensity of Rac1 and DH domain to bind together. Indeed, it is well known that during their interaction, the proteins are not rigid bodies but undergo conformational changes to reach the most favorable arrangement (Goh et al., 2004). The second region analyzed was α3-5, which was closer to the PH domain in AlsinBnd than AlsinUnBnd (Figure 5A). Hence, it is possible that an interaction between α3-5 and PH domain plays a role in the stabilization of Alsin open conformation.

To summarize, this study highlights that free Alsin DH/PH domain exists mainly in a closed state, where the interaction between RLD and C-terminus may hide the putative dimerization surface. In these conformations, the helix α6 is bent such that its interaction with Rac1 might be unlikely. When, due to thermal energy, the PH domain moves to reach a more open state and helix α6 curvature decreases, the DH domain may be more prone to bind Rac1. Such interaction, together with the one between α3-5 and PH, seems to stabilize a linear conformation of DH/PH domain. At the same time, dimerization through DH domain may be favored by Rac1 interaction since it reduces the fluctuations within the residues that should mediate such processes.

## Conclusion

The understanding of molecular mechanisms underlying Alsin biological functions is still limited, and therefore, a clear comprehension of molecular mechanisms driving ALS2-related pathologies is difficult. In this view, investigating nanoscale phenomena involved in Alsin-mediated physiological pathways may shed light on the structure–function relationships and provide insight on essential mechanisms involved in neuronal homeostasis, whose impairment may lead to diseases such as IAHSP. However, the Alsin structure has not been resolved yet. In this framework, the first step is represented by the development of Alsin 3D atomistic model and the analysis of its dynamics. This work provides an all-atom, 3D structure for the DH/PH domain of Alsin, refined in explicit water, by molecular modeling techniques, available for the scientific community.

The analysis on Alsin DH/PH region, both with and without Rac1, revealed that its CR1 should not be involved in the interaction with its binding partner, unlike other Rho GEF proteins. This result suggests a possible molecular basis of its different biological functions. Moreover, the mechanism by which Rac1 may induce conformational changes that trigger the membrane relocalization has been investigated. While stabilizing a more open and linear conformation of the whole domain by changing the DH–PH relative position, the interaction with Rac1 caused a local increase in the mechanical properties within the putative dimerization site. As hypothesized from previous literature (Sato et al., 2018), Rac1 interaction could induce conformational changes on the RLD domain. Results of this study suggest that Rac1 also affects DH/PH conformation which may also participate in the unlocking of the RLD-mediated inhibitory switch process. Further *in silico* investigations and additional *in vitro* analysis will be needed to elucidate mechanisms and relationships between Rac1, DH/PH, and RLD.

## Data Availability Statement

The original contributions presented in the study are included in the article/Supplementary Material, further inquiries can be directed to the corresponding author.

## Author Contributions

MM and MCn carried out molecular dynamics simulations and data analysis. MD was responsible for the research supervision. All authors have contributed to the conceptualization, data rationalization, manuscript writing, and read and approved the final manuscript.

## Conflict of Interest

The authors declare that the research was conducted in the absence of any commercial or financial relationships that could be construed as a potential conflict of interest.

## Publisher’s Note

All claims expressed in this article are solely those of the authors and do not necessarily represent those of their affiliated organizations, or those of the publisher, the editors and the reviewers. Any product that may be evaluated in this article, or claim that may be made by its manufacturer, is not guaranteed or endorsed by the publisher.

## Footnotes

1
https://alphafold.ebi.ac.uk/entry/Q96Q42
