# Supplementary material for: Prediction of Protein–Protein Interactions Between Alsin DH/PH and Rac1 and Resulting Protein Dynamics
Source: Front Mol Neurosci. 2022 Jan 20;14:772122. doi: 10.3389/fnmol.2021.772122 (PMC8811474; doi:10.3389/fnmol.2021.772122)
Supplement: Supplementary file 1 [file Data_Sheet_1.PDF]

Prediction of protein-protein interactions between Alsin DH/PH and Rac1 and resulting protein dynamics

Running Title: Alsin DH/PH-Rac1 interaction mechanisms

Marco Cannariato[1], Marcello Miceli[1**], Marco Cavaglià[1], Marco A. Deriu[1*]**

[1] PolitoBIOMed Lab, Department of Mechanical and Aerospace Engineering, Politecnico di Torino, 10129 Turin, Italy

[**]These authors equally contributed to this study

***Correspondence:**
marco.deriu@polito.it

Supplementary Material 1 (SM1)

SM1 1 Introduction

Ras homologue (Rho) are a family of GTPases involved in many cellular functions linked to the dynamics of actin cytoskeleton (Wennerberg et al., 2005). Their activity is regulated also by Rho guanine-nucleotide exchange factors (GEF), whose structure is characterized by a Dbl-homology (DH) domain followed by a Pleckstrin-homology (PH) domain. The dynamics of some Rho GEFs has been analysed to investigate the relationship between their structure, its evolutionary-driven deformations, and their biological functions (Raimondi et al., 2015; Felline et al., 2019). To strengthen the results obtained from the analysis of Alsin DH/PH domain, the experimental setup used in our molecular dynamics (MD) simulations has been validated reproducing previous results from literature on LARG, a known Rho GEF, both in presence and in absence of its binding partner RhoA (Felline et al., 2019). Moreover, the interaction between LARG and RhoA has been further investigated and compared to the one between Alsin and Rac1 to shed light on the different biological functions of the two DH/PH domains. Indeed, Alsin is a Rac1 effector rather than GEF (Kunita et al., 2007).

SM1 2 Materials and Methods

SM1 2.1 Molecular Dynamics

The structures of LARG alone (PDB: 1TXD) and bound to RhoA (PDB: 1X86) were retrieved from the Protein Data Bank (Kristelly et al., 2004). For consistency with previous literature residues have been numbered according to UniProt entry Q9NZN5. Two MD simulations were performed, one for the protein without the GTPase ($LARG^{UnBnd}$) and one for the protein bound to RhoA ($LARG^{Bnd}$), using GROMACS 2020.4 (Lindahl et al., 2020). AMBER ff99SB-ILDN force field was used to define the topology (Lindorff-Larsen et al., 2010). Both systems were configured in GROMACS in a cubic box with periodic boundary conditions setting a minimum distance of 1 nm between the protein and the box edge. Then, they were solvated in explicit TIP3P water (Jorgensen et al., 1983) and, subsequently,

an appropriate number of Na^+ and Cl^- were added to reach a physiological concentration of 0.15 M and to neutralize the charge. The energy minimization was performed through the steepest descend method for 2000 steps before equilibrating the systems. To this purpose, the following procedure was performed in both of them. An initial simulation of 500 ps in NVT ensemble and a following one of 500 ps in NPT ensemble were carried out restraining C-alpha carbons positions. The NVT simulation was performed with position restraints at a reference temperature of 300 K using the modified Berendsen thermostat (Berendsen et al., 1984) with $\tau = 0.1$ ps. The NPT simulation was carried out at 1.0 bar under position restraints using the Berendsen barostat with isotropic coupling and $\tau = 1.0$ ps. Finally, an MD simulation in NPT ensemble was produced for 260 ns, 10 ns of pre-equilibration followed by 250 ns of dynamics. The equation of motion was integrated with the leap frog algorithm using a time step of 2 fs. Electrostatic interactions were treated with particle mesh Ewald method, short-range cut-off at 1.2 nm and a switching of the potential starting at 1.0 nm. Van der Waals interaction were treated with a cut-off at 1.2 nm and a switching of the potential starting at 1.0 nm. The Visual Molecular Dynamics (VMD) engine was used for the visual inspection of systems and trajectories (Humphrey et al., 1996).

SM1 2.2 Analysis

The stability of each system was evaluated computing the root-mean-square deviation (RMSD) from the initial configuration of C-alphas atomic positions throughout the trajectory. Since the dynamics of the protein has been previously described as characterized by a collective motion of PH domain, the RMSD was also evaluated on the C-alphas of the sole DH region (residues 766-996). From the visual inspection of RMSD plots (FigureSM1 1), last 240 ns of each trajectory was used in the following analysis. The flexibility of the protein was evaluated computing the root-mean-square fluctuation (RMSF) during the last 240 ns and fitting the structures on the C-alphas of the DH domain. The mechanical properties at the residue level were inferred computing the force constant profile. The distances were defined between the C-alphas of the amino acids and computed on representative snapshots extracted every 50 ps. The force constants were computed independently for the DH domain (residues 766-996) and the following region (residues 997-1126), comprising PH domain and the linker region.

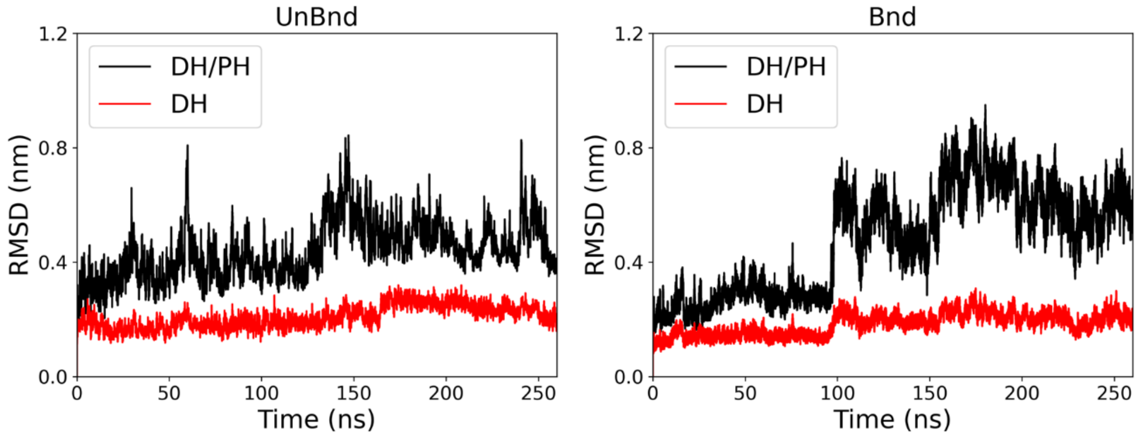

FigureSM1 1. RMSD plots of LARG^{UnBnd} and LARG^{Bnd}.

To identify LARG residues involved in the interaction with RhoA, the probability to be in contact with RhoA was computed for each amino acid of LARG^{Bnd}. The contact probability was computed sampling the MD trajectory every 250 ps with the following procedure (Deriu et al., 2014). For each sample snapshot, the distances between the atoms of one LARG residue and the atoms of RhoA were computed: the residue was in contact if at least one of the residue-residue distances was lower than a threshold of 0.3 nm. The number of snapshots in which a residue was in contact divided by the total number of snapshots was the contact probability for that residue. Principal component analysis (PCA) was performed in both systems to analyse whether the presence of RhoA alters the essential dynamics of the RhoGEF. The covariance matrix was built on the C-alphas using the ones of DH domain for least square fit as done before (Felline et al., 2019).

GROMACS built-in tools have been used to compute RMSD, RMSF, and to perform PCA, while the calculation of contact probabilities and force constants was implemented using python libraries and custom scripts (Michaud-Agrawal et al., 2011; Gowers et al., 2016).

SM1 2.3 Plots and Figures

Three-dimensional representations of the proteins were rendered in VMD. The principal directions were depicted through porcupine plots obtained from a custom made VMD script. In the porcupine plots, each C-alpha is associated to a segment oriented along the principal direction. The length of such segments is proportional to the amplitude of fluctuations along the represented direction. Data plots for RMSD, RMSF, force constants, and contact probability were generated using python libraries (Hunter, 2007).

SM1 3 Results

SM1 3.1 RhoA interaction and mechanical properties

Four main regions were involved in the interaction between LARG and the GTPase (FigureSM1 2). Helices $\alpha1$ and $\alpha5$, corresponding to the first conserved region (CR1) and the third conserved region (CR3), were characterized by a high probability to be in contact with RhoA, supporting previous findings that those regions are crucial in the GEF activity of this family of proteins. Moreover, the central part of the helix $\alpha6$ interacted with RhoA through almost all the dynamics suggesting its role in the regulation of the GTPase catalytic activity. Finally, there were some residues in helices $\alpha3$ and

$\alpha 4$ and in the non-structured region between $\alpha 4$ and $\alpha 5$ with a high contact probability, therefore these regions may have an auxiliary function in RhoA binding. Notably, PH domain did not show amino acids with a significant probability to be involved in the interaction despite being close to RhoA in the starting configuration.

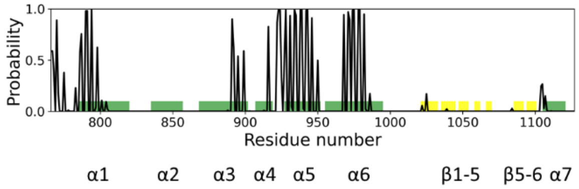

FigureSM1 2. Contact probability (bottom) and mechanical profile (top) of LARG. The secondary structure of DH and PH domains is highlighted to emphasize $\boldsymbol{\alpha}$-helices (green) and $\boldsymbol{\beta}$-strands (yellow).

Previously, the mechanical profile of this protein was investigated. Independently of the functional state, the higher values of the force constants were located within the structured regions. Moreover, the peaks of the profiles corresponded to highly conserved residues within DH/PH domains. Finally, the presence of RhoA increased on average the mechanical rigidity of the protein. The same analysis was performed to investigate whether the different experimental setup could have had an influence on the dynamics of LARG. The force constants obtained from our MD simulations were comparable to the ones from literature, both in the unbound and in the bound states (FigureSM1 3). Moreover, in line with the previous findings, the mechanical profile of UnBnd and Bnd systems was similar.

Independently of RhoA presence, the residues with the highest force constants were located in the helix $\alpha 5$. Here, the interaction with RhoA induced a remarkable increase of rigidity in the Bnd system, especially evident in the N-terminus of the region. The same effect could be observed within helix $\alpha 1$, except for the C-terminus where the values were comparable in the two functional states of LARG. The presence of RhoA did not cause significant differences within the last part of helix $\alpha 3$, while induced only a slight reduction in the fluctuations of helix $\alpha 4$. Moreover, helix $\alpha 6$ was characterized by low force constant values with no difference between the Bnd and UnBnd states despite interacting with RhoA. Therefore, the greatest change in the mechanical profile of the protein when it bound RhoA was represented by an increase of force constants within CR1 and CR3, which are crucial for the GEF activity of LARG. Despite showing some different values, on average the profile was similar to the one previously obtained and the main alterations due to the interaction were located in the same structured regions.

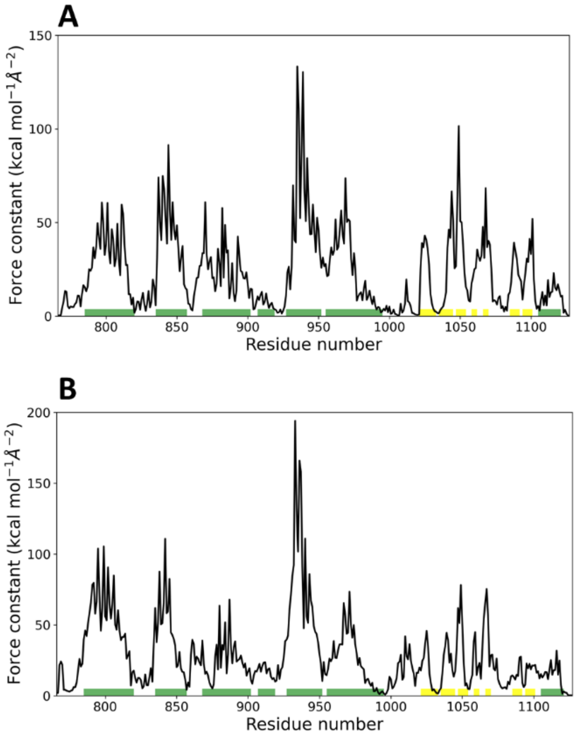

FigureSM1 3. Force constant profile of **(A)** LARG^{UnBnd} and **(B)** LARG^{Bnd}. The secondary structure of DH and PH domain is highlighted to emphasize $\boldsymbol{\alpha}$-helices and $\boldsymbol{\beta}$-strands in green and yellow, respectively.

The flexibility of LARG in the two states was initially evaluated through the RMSF on C-alphas. To compute this measure, protein structures were fitted on the C-alphas of the DH domain as was done previously in literature (Raimondi et al., 2015; Felline et al., 2019). The analysis on a similar system had revealed that the most flexible part of the protein is located mainly in the region following DH domain, with peaks corresponding to the loops connecting the β-strands of PH domain and the one linking DH and PH domains. Moreover, the fluctuations of the protein in two functional states were comparable with only a slightly increased mobility of free LARG. According to the results on these systems, the RMSF profile revealed that the two functional states have comparable flexibilities, but the Bnd system showed slightly greater fluctuations. In both cases, the essential motion resides in PH domain and the linker region, with the involvement of the last residues of helix α6. Moreover, it was possible to observe a reduction in the motion of helix α4 and the subsequent loop in the Bnd system, in agreement with the previous findings of its interaction with RhoA. Despite small differences, the flexibility profiles of these systems were similar to the ones previously obtained, meaning that the different experimental setup had not influenced LARG dynamics, independent of the functional state (FigureSM1 4).

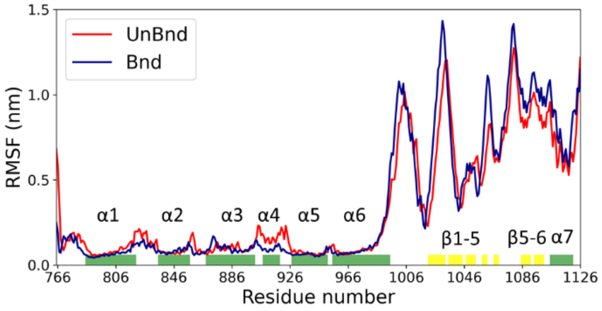

FigureSM1 4. Comparison between the flexibility profile of LARG^{UnBnd} and LARG^{Bnd}. The secondary structure of DH and PH domains is highlighted to emphasise $\boldsymbol{\alpha}$-helices and $\boldsymbol{\beta}$-strands in green and yellow, respectively.

SM1 3.2 Analysis of the dynamics

To understand the essential dynamics that characterizes RhoGEFs, PCA has been applied to MD trajectories of several RhoGEF oncoproteins characterized by the DH/PH structure. This analysis had shown that, in all functional states, more than 80% of the total variance was represented by the first two principal components (FigureSM1 5), which describe a collective motion of PH domain and the terminal region of helix α6. Moreover, there was a significant overlap between these two directions between the bound and unbound states of the same protein (Raimondi et al., 2015). Here, PCA has been used to investigate whether such collective motions were detectable also in these systems. In agreement with the previous findings, the first two principal components (PC1 and PC2) explained almost the totality of the variance. In both functional states, PC1 and PC2 represented two different rotational motions of PH domain, together with the linker region and the last residues of DH domain (FigureSM1 5). As previously observed, there was a remarkable overlap between the principal components of the two functional states. However, these results showed that, while in the UnBnd system PH domain moved closer to RhoA binding surface, in the Bnd system the dynamics followed the same directions but in the opposite way.

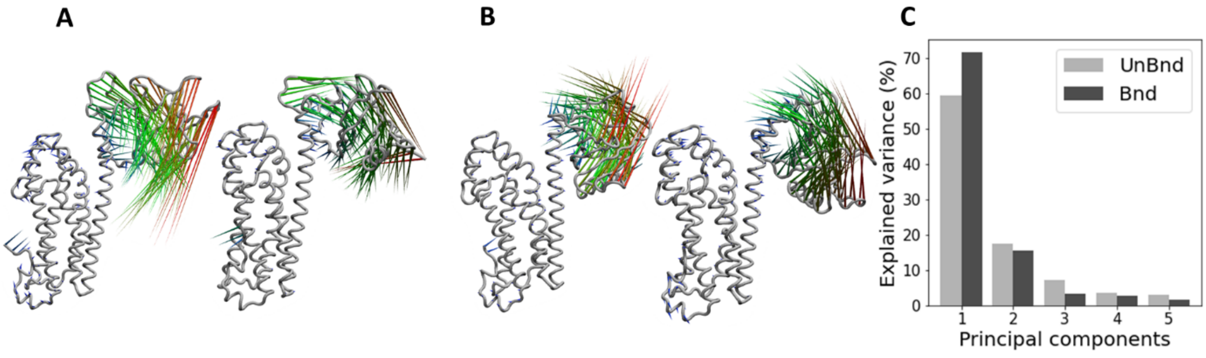

FigureSM1 5. Principal component analysis of LARG dynamics. **(A)** Comparison between the first (left) and second (right) principal components of LARG^{UnBnd}. **(B)** Comparison between the first (left)

and second (right) principal components of $LARG^{Bnd}$. **(C)** Explained variances of the first five principal components.

SM1 4 Discussion

The dynamics of a known RhoGEF protein has been analysed in its free and RhoA-bound form and the results have been compared to previous findings on similar systems (Felline et al., 2019). The close affinity of the obtained results with earlier literature provided a strong proof that the employed force field was able to model the dynamics of proteins characterized by the DH/PH motif. Indeed, the mechanical profile of LARG has been reproduced and force constants values were similar to the ones previously obtained (FigureSM1 3). The presence of RhoA increased on average the mechanical rigidity of the protein, especially in the regions involved in the interaction and in the loop connecting the DH and PH domains. Moreover, the C-terminal part of helix $\alpha6$ was characterized by low rigidity independent of the functional state of LARG. In agreement with previous literature, the essential dynamics of this protein was a collective motion of PH domain, the region linking DH and PH regions, and the last part of helix $\alpha6$ (FigureSM1 4). The amount of fluctuation was similar in the bound and unbound states, meaning that the flexibility of the protein was not altered by the presence of its ligand partner. While previously slightly greater flexibility has been observed for free LARG, in the studied systems the PH domain was lightly more mobile in the bound form. Finally, it was possible to observe increased fluctuations in the region around helix $\alpha4$ in absence of RhoA. Moreover, the PCA has showed that the essential dynamics could be described through two different roto-translational motions of PH domain. In fact, around 80% of the total variance could be expressed by the two first principal components (FigureSM1 5). Notably, these directions were not altered by the presence of RhoA. However, the versus of motion in the Bnd system was opposite to the one in UnBnd system, while this difference was not observed before.

The information about regions involved in the interaction with RhoA given by the crystallographic structure is only partial. Indeed, the arrangement of proteins in a crystal may differ from the one in solution (Acharya and Lloyd, 2005) and the dynamic information of the interaction is lost. Therefore, the probability for each residue of LARG to be in contact with RhoA was investigated throughout the MD simulation (FigureSM1 2). Despite being close in the initial configuration, the probability of having a contact between residues in PH domain and RhoA were very limited. The residues with highest probability to interact with the GTPase were located in CR1 and CR3, in agreement with their role in the GEF activity of this family of proteins. Besides, helices $\alpha3$ and $\alpha4$ might have had a role in stabilizing the interaction of RhoA with LARG due to the presence of residues with high contact probability.

To conclude, the differences between the obtained results and the previous ones are contained within the range of variability of the employed methods. Moreover, the evidences were reproduced faithfully carrying out only one MD simulation instead of three, as done previously. Hence, the close similarity represented a validation of the employed experimental setup and the proof that it could be exploited for further analysis on Alsin DH/PH domain in order to strengthen the obtained results.

Reference

Acharya, K. R., and Lloyd, M. D. (2005). The advantages and limitations of protein crystal structures. *Trends Pharmacol. Sci.* 26, 10–14. doi:10.1016/j.tips.2004.10.011.

Berendsen, H. J. C., Postma, J. P. M., van Gunsteren, W. F., DiNola, A., and Haak, J. R. (1984). Molecular dynamics with coupling to an external bath. *J. Chem. Phys.* 81, 3684–3690. doi:10.1063/1.448118.

Deriu, M. A., Grasso, G., Licandro, G., Danani, A., Gallo, D., Tuszynski, J. A., et al. (2014). Investigation of the Josephin Domain Protein-Protein Interaction by Molecular Dynamics. *PLoS One* 9, e108677. doi:10.1371/journal.pone.0108677.

Felline, A., Belmonte, L., Raimondi, F., Bellucci, L., Fanelli, F., Felline, A., et al. (2019). Interconnecting flexibility , structural communication , and function in RhoGEF oncoproteins. doi:10.1021/acs.jcim.9b00271.

Gowers, R., Linke, M., Barnoud, J., Reddy, T., Melo, M., Seyler, S., et al. (2016). MDAnalysis: A Python Package for the Rapid Analysis of Molecular Dynamics Simulations. in, 98–105. doi:10.25080/Majora-629e541a-00e.

Humphrey, W., Dalke, A., and Schulten, K. (1996). VMD - Visual Molecular Dynamics. *J. Mol. Graph. Model.* 14, 33–38.

Hunter, J. D. (2007). Matplotlib: A 2D Graphics Environment. *Comput. Sci. Eng.* 9, 90–95. doi:10.1109/MCSE.2007.55.

Jorgensen, W. L., Chandrasekhar, J., Madura, J. D., Impey, R. W., and Klein, M. L. (1983). Comparison of simple potential functions for simulating liquid water. *J. Chem. Phys.* 79, 926–935. doi:10.1063/1.445869.

Kristelly, R., Gao, G., and Tesmer, J. J. G. (2004). Structural Determinants of RhoA Binding and Nucleotide Exchange in Leukemia-associated Rho Guanine-Nucleotide Exchange Factor. *J. Biol. Chem.* 279, 47352–47362. doi:10.1074/jbc.M406056200.

Kunita, R., Otomo, A., Mizumura, H., Suzuki-Utsunomiya, K., Hadano, S., and Ikeda, J. E. (2007). The Rab5 activator ALS2/alsin acts as a novel Rac1 effector through Rac1-activated endocytosis. *J. Biol. Chem.* 282, 16599–16611. doi:10.1074/jbc.M610682200.

Lindahl, Abraham, Hess, and Spoel, van der (2020). GROMACS 2020.4 Source code. doi:10.5281/ZENODO.4054979.

Lindorff-Larsen, K., Piana, S., Palmo, K., Maragakis, P., Klepeis, J. L., Dror, R. O., et al. (2010). Improved side-chain torsion potentials for the Amber ff99SB protein force field. *Proteins Struct. Funct. Bioinforma.* 78, 1950–1958. doi:10.1002/prot.22711.

Michaud-Agrawal, N., Denning, E. J., Woolf, T. B., and Beckstein, O. (2011). MDAnalysis: A toolkit for the analysis of molecular dynamics simulations. *J. Comput. Chem.* 32, 2319–2327. doi:10.1002/jcc.21787.

Raimondi, F., Felline, A., and Fanelli, F. (2015). Catching Functional Modes and Structural Communication in Dbl Family Rho Guanine Nucleotide Exchange Factors. *J. Chem. Inf. Model.* 55, 1878–1893. doi:10.1021/acs.jcim.5b00122.

Wennerberg, K., Rossman, K. L., and Der, C. J. (2005). The Ras superfamily at a glance. *J. Cell Sci.* 118, 843–846. doi:10.1242/jcs.01660.
