# Supplementary material for: Prediction of Protein–Protein Interactions Between Alsin DH/PH and Rac1 and Resulting Protein Dynamics
Source: Front Mol Neurosci. 2022 Jan 20;14:772122. doi: 10.3389/fnmol.2021.772122 (PMC8811474; doi:10.3389/fnmol.2021.772122)
Supplement: Supplementary file 2 [file Data_Sheet_2.PDF]

Prediction of protein-protein interactions between Alsin DH/PH and Rac1 and resulting protein dynamics

Running Title: Alsin DH/PH-Rac1 interaction mechanisms

Marco Cannariato[1], Marcello Miceli[1**], Marco Cavaglià[1], Marco A. Deriu[1*]**

[1] PolitoBIOMed Lab, Department of Mechanical and Aerospace Engineering, Politecnico di Torino, 10129 Turin, Italy

[**]These authors equally contributed to this study

***Correspondence:**
marco.deriu@polito.it

Supplementary Material (SM2)

SM2 1 Conformational Stability

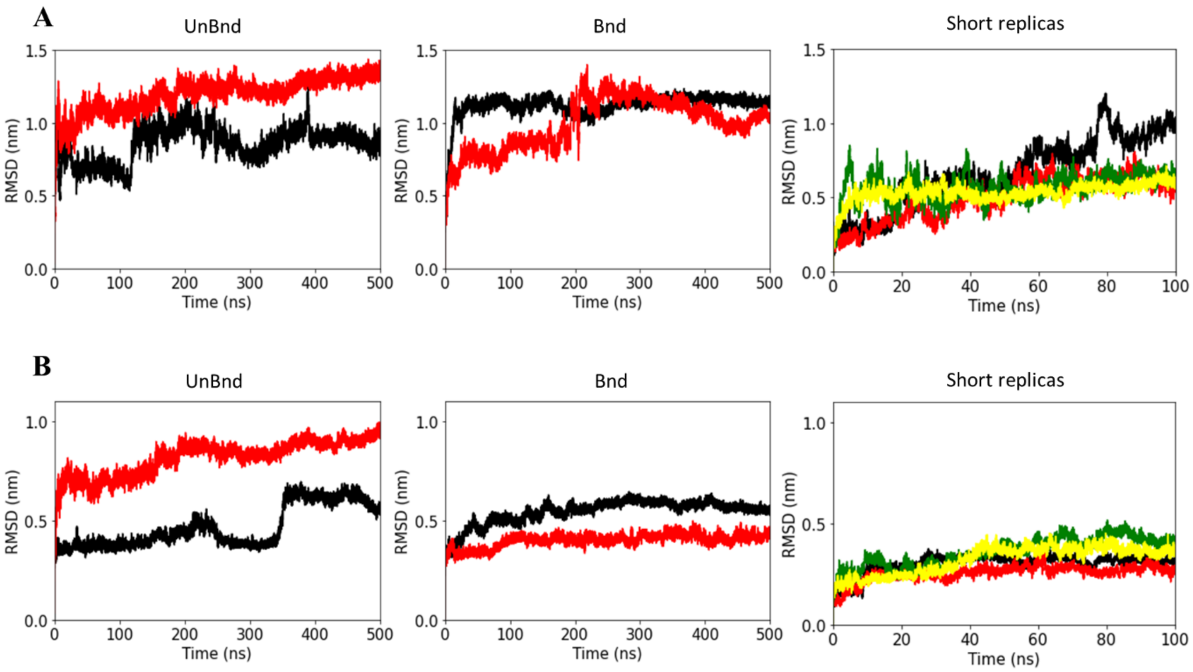

FigureSM2 1. RMSD plots of **(A)** protein C-alphas and **(B)** DH domain C-alphas from the initial configuration. For the sake of clarity, different colours are used to represent different replicas.

SM2 2 Mechanical properties

The flexibility of the protein was evaluated by computing the root-mean-square fluctuation (RMSF) during the last 450 ns of long replicas and fitting the structures on the C-alphas of the DH domain. Previously, the force constant per residue profile was investigated homologous domains to infer its mechanical properties at the single residue level. This is a measure of the fluctuations of the mean distance of each residue from the rest of the structure and its higher values have been associated with protein functional sites (Navizet et al., 2004; Lavery and Sacquin-Mora, 2007). The calculation of force constants was implemented according to the formula:

$$k_i = \frac{3k_B T}{\langle (d_i - \langle d_i \rangle)^2 \rangle} \tag{1}$$

where d_i is the mean distance of the i-th residue from the rest of the structure, $k_B$is the Boltzmann's constant, T is the temperature of the system, and the operator $\langle \, \rangle$ stands for the average over the simulation. The distances were defined between the C-alphas of the amino acids and computed on representative snapshots extracted every 50 ps. The force constants were computed independently for the DH domain (residues 766-996) and the following region (residues 997-1126), comprising the PH domain and the linker region (Sacquin-Mora et al., 2007).

SM2 3 Helix axis curvature

To investigate the curvature of $\alpha 6$ helix, representative snapshots were extracted every 50 ps for both long and short replicas. For each snapshot, the x and z coordinates of the alpha carbons were picked, then the centres of mass of successive groups of four C-alphas were considered as points of the helix axis. Therefore, the i-th sample of the axis is obtained selecting from the i-th to the *(i+3)*-th alpha carbons and computing their centre of mass. The obtained points were interpolated, using the x coordinate as an independent variable, with a second-degree polynomial function $c(x)$ which was then evaluated in 100 points to approximate the helix axis. Finally, the curvature $\kappa(x)$ and the integral of the curvature I_κ were computed as:

$$\kappa(x) = \frac{|c''(x)|}{(1 + c'(x)^2)^{\frac{3}{2}}} \qquad I\kappa = \int \kappa(x)\, dx \tag{2}$$

The integral was numerically solved using the composite trapezoidal rule. Higher values of curvature integral are related to higher deviations from the straightness of the helix axis.

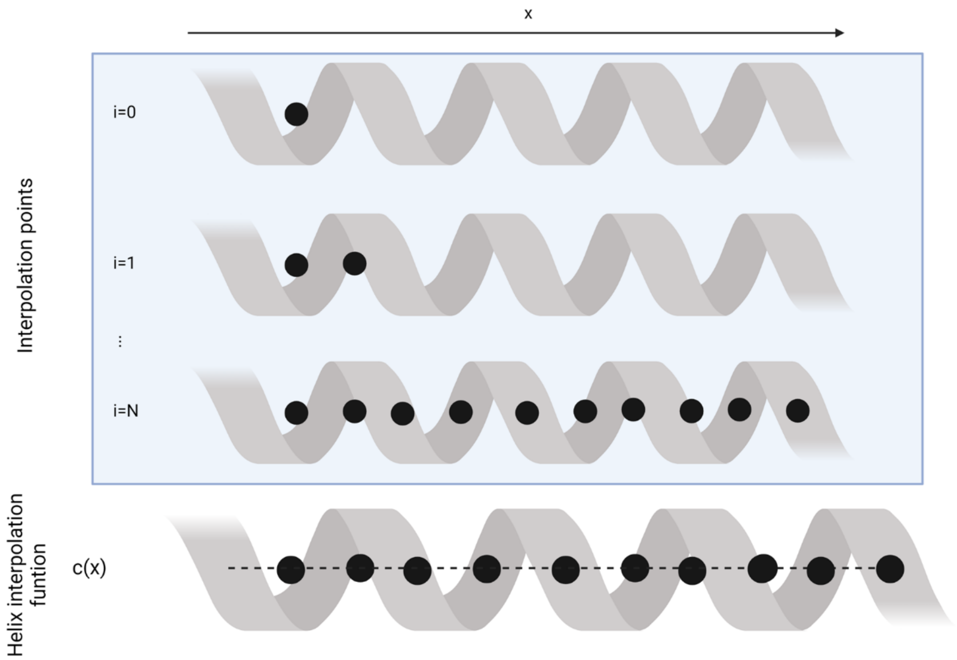

FigureSM2 2. Approximation of the helix axis. Successive groups of four atoms are considered and the centre of mass is computed for each of them. Then, the axis is represented by a second-degree polynomial function $\boldsymbol{c}(\boldsymbol{x})$ obtained through the interpolation of the obtained points.

SM2 4 Validation of the Markov State Model

The validation of a Markov State Model is usually done through the Chapman-Kolmogorov test. It tests whether the chosen discretization and lag time have led to a model that satisfies the approximation of the Chapman-Kolmogorov equation within statistical uncertainty. If $[T(\tau)]^k$ is the k-fold application of the transition matrix of the model to validate, i.e. the matrix multiplied by itself k times, and $T(k\tau)$ the transition matrix at the greater lag time $k\tau$, the approximation:

$$T(k\tau) \approx [T(\tau)]^k$$

was tested estimating $T(k\tau)$ through a *Markov chain Monte Carlo sampling* of 100 transition matrices from 2 independent Markov chains. In particular, for a given lag time the mean of the obtained distribution was used as a transition matrix and the standard deviation was taken as a measure of statistical uncertainty. The test was performed for $k > 2$ and $k\tau < 50$ ns to avoid excessive under-sampling of short trajectories.

SM2 5 Homology modelling of Alsin DH/PH domain

TableSM2 1. Information about the templates used to build Alsin homology model**.**

PDB	Identity (%)	Similarity (%)	Ramachandran outliers (%)
1FOE (Worthylake et al., 2000)	16.9	29.2	2.5
1KI1 (Snyder et al., 2002)	12.9	29.2	3.3
1NTY (Skowronek et al., 2004)	10.5	26.5	0.0
1XCG (Derewenda et al., 2004)	14.2	28.3	1.5
1X86 (Kristelly et al., 2004)	13.5	29.8	0.3
2DFK (Xiang et al., 2006)	10.2	27.7	0.6
2PZ1 (Mitin et al., 2007)	11.4	30.5	2.4
2RGN (Lutz et al., 2007)	10.5	25.8	0.3
2Z0Q (Murayama et al., 2012)	13.2	28.9	0.0
3MPX (Shen et al.)	10.5	22.5	0.0
3ODO (Chen et al., 2011)	14.2	28.9	0.9
4D0N (Abdul Azeez et al., 2014)	12.0	31.1	0.0
4GZU (He et al., 2013)	11.4	24.6	0.0
4XH9 (Petit et al., 2018)	12.9	28.9	0.3
4YON (Lucato et al., 2015)	14.8	32.3	0.0
6D8Z (Bandekar et al., 2019)	11.1	26.5	0.0

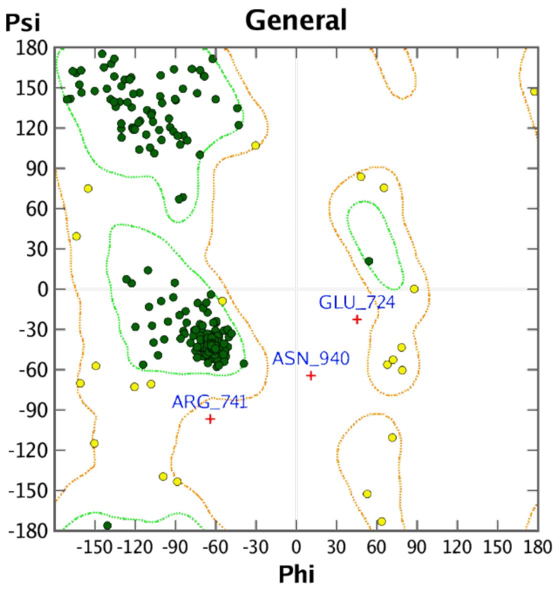

FigureSM2 3. Ramachandran plot of Alsin DH/PH homology model. Residues in the core regions, residues in allowed regions, and outliers are coloured in green, yellow, and red, respectively.

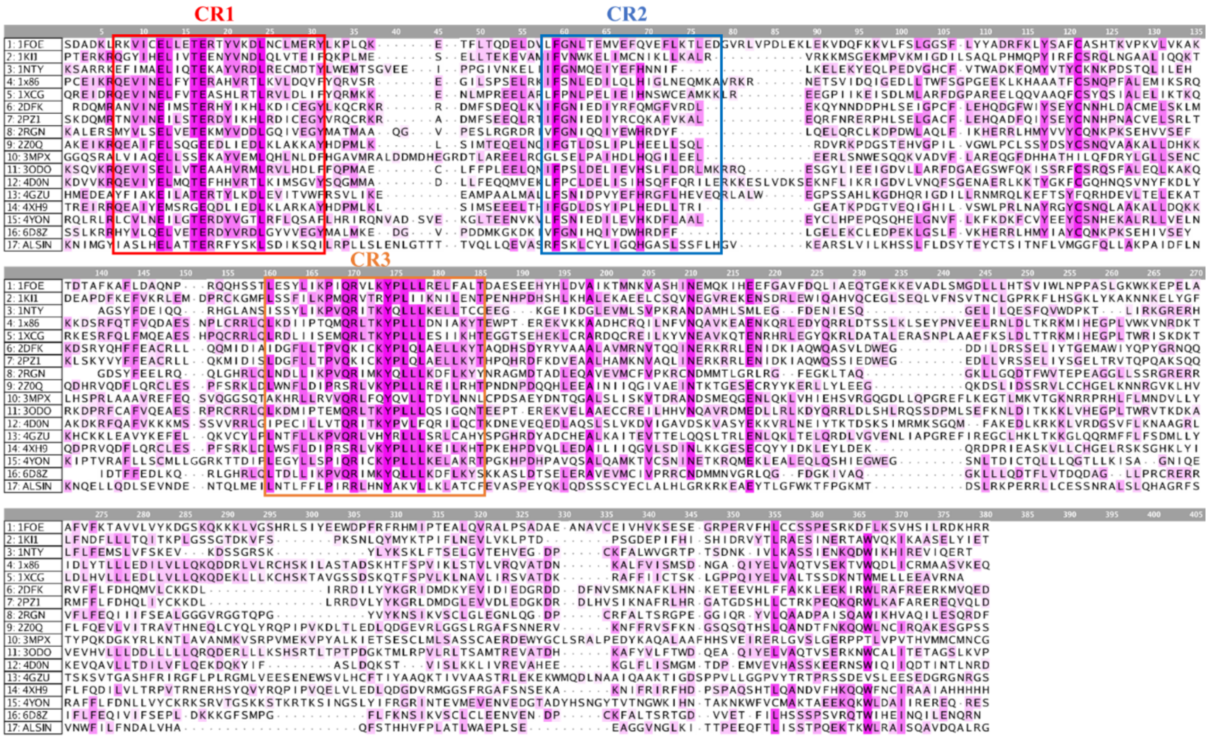

FigureSM2 4. Alignment of Alsin and the 16 templates used by I-Tasser to build the homology model. The templates residues that were not aligned with Alsin sequence are not included.

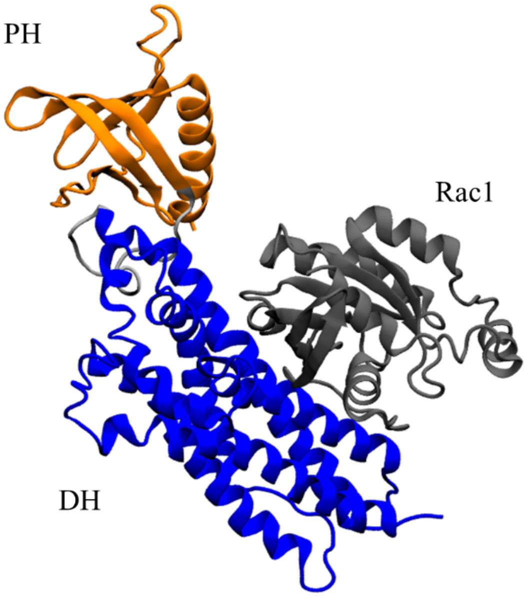

FigureSM2 5. Initial configuration of Alsin^{Bnd}.

TableSM2 2. List of residues characterized by high contact probability, high conservation, or an increased force constant due to Rac1 interaction. The level of conservation of each amino acid has been inferred through ConSurf web server (https://consurf.tau.ac.il/) using the multiple sequence alignment exploited to identify Alsin conserved regions (Ashkenazy et al., 2016). Residues sharing all three attributes are highlighted in yellow.

Condition	Residue number
In contact[a]	I789 T790 F793 F838 I841 R842 H845 A879 L882 R886
Conserved[b]	Y691 S694 L695 E697 L698 T701 E702 F705 Y706 K708 L709 I716 L720 E737 F742 7K44 L745 L748 H752 756 759 764 781 I789 792 795 796 797 798 799 802 806 819 824 825 833 835 837 F838 840 I841 R842 R843 L844 H845 N846 Y847 A848 K849 V850 L851 L854 F858 S862 E864 S871 Y875 L878 L882 G883 K885 R886 E890 Y891 G894 F895 F899 M903 R912 F936 A961 E966 L985 S987 T989 K993 W996 I1000
Increased force constant[c]	H696 E697 L698 A699 T701 F705 S707 K708 G750 Q751 G753 A754 S755 S757 S758 F759 L760 H761 K773 H774 S776 L816 E831 N834 N846 K849 L882 R886 Q947 L959 P979

[a]A residue is considered in contact with Rac1 if the contact probability is greater or equal to 0.9

[b]A residue is considered conserved if the ConSurf conservation is greater or equal to 7

[c]To identify the residues with increased force constant due to Rac1-interaction, the following procedure was performed. First, high mechanical rigidity residues were identified for the bound and unbound states as the ones with force constants greater than the average of the profile. Then, the sets of residues were compared to find those identified in the Alsin^{Bnd} and not in Alsin^{UnBnd}.

SM2 6 Flexibility profile of Alsin DH/PH domain

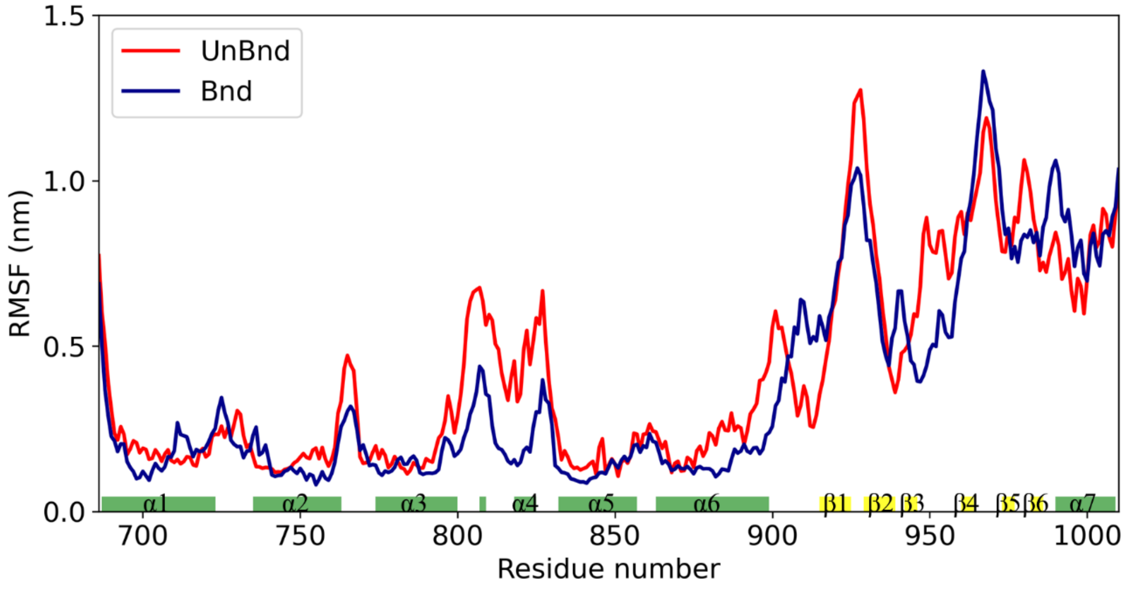

FigureSM2 6. RMSF of free and Rac1-bound Alsin. Helices and strands are represented in green and yellow, respectively.

SM2 7 Markov State Model of Alsin^{UnBnd}

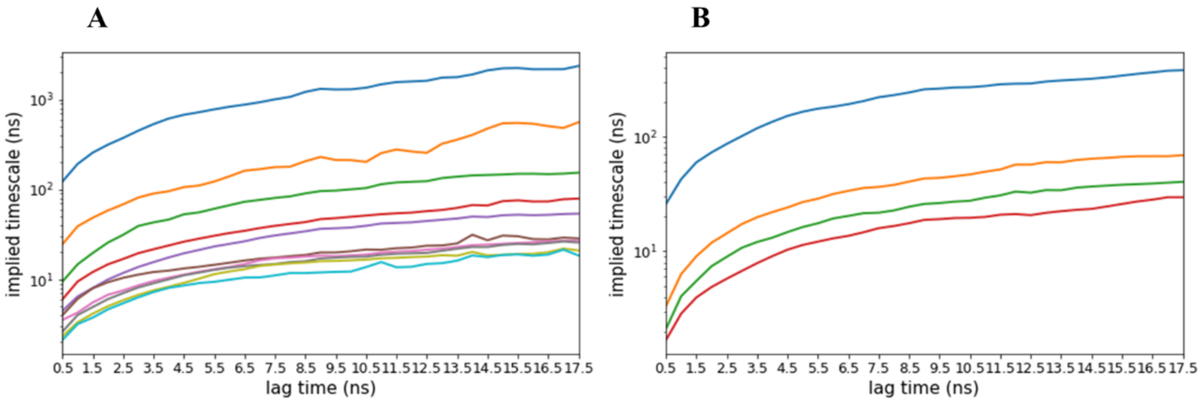

FigureSM2 7. Implied timescales plot using the discretization in **(A)** 1000 clusters from K-Centres and **(B)** 5 states obtained through PCCA+.

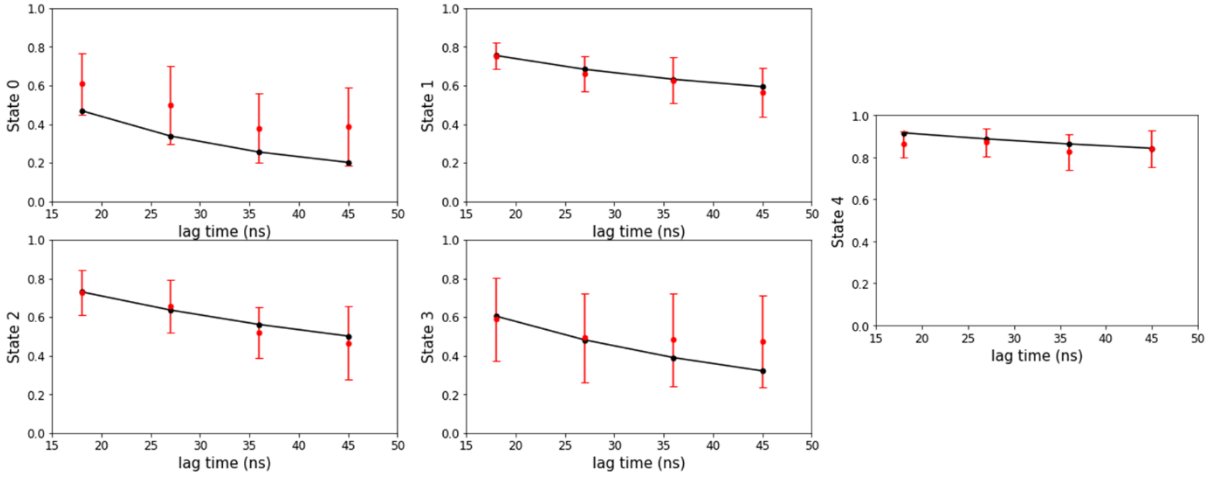

FigureSM2 8. Chapman-Kolmogorov test of the Markov State Model. Only self-transition probabilities are represented. The black line represents the transition probability estimated propagating the MSM, red points and error bars represent the mean and standard deviation of the transition probability at multiples of the lag time estimates through the Bayesian approach.

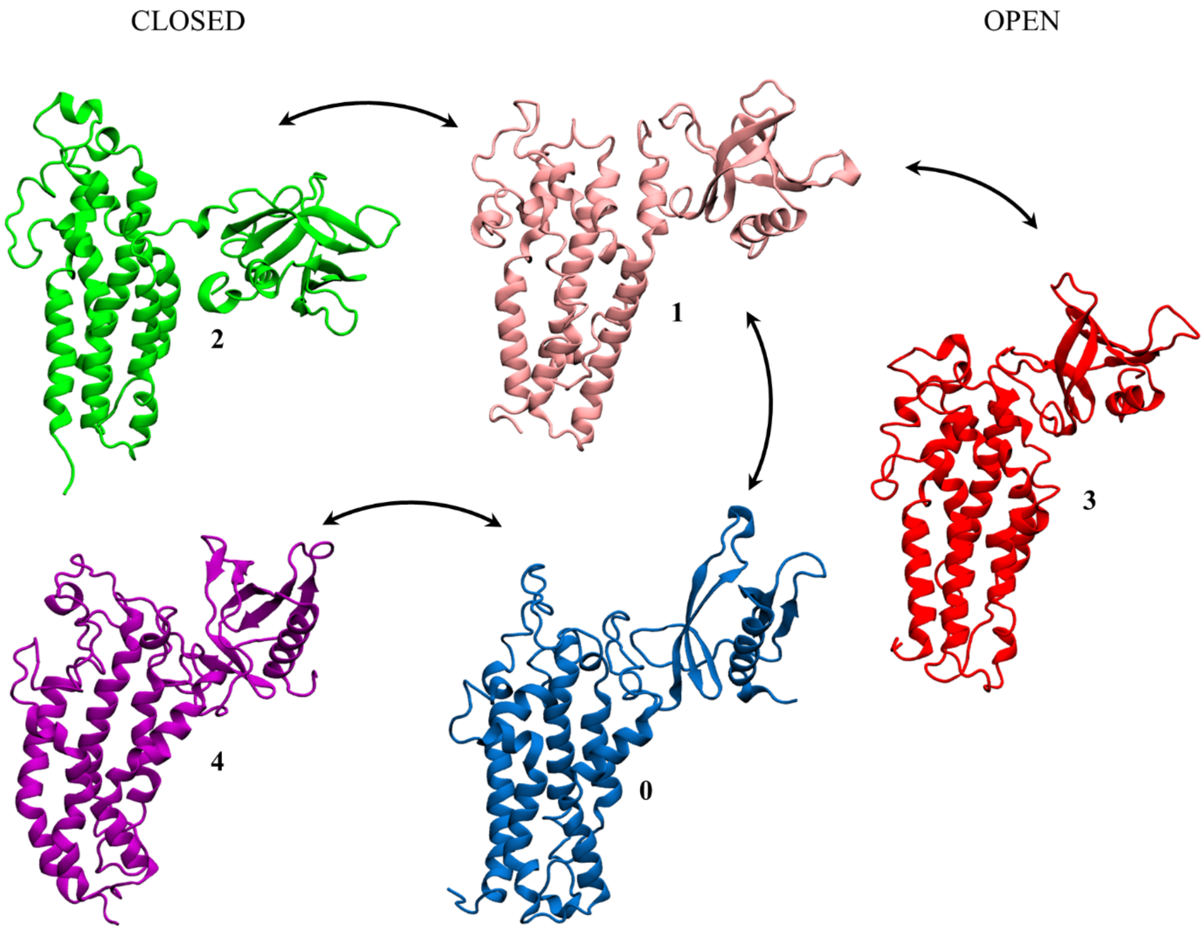

FigureSM2 9. Representation of the most probable transition pathways of Alsin^{UnBnd} between the open to the closed states.

Reference

Abdul Azeez, K. R., Knapp, S., Fernandes, J. M. P., Klussmann, E., and Elkins, J. M. (2014). The crystal structure of the RhoA–AKAP-Lbc DH–PH domain complex. *Biochem. J.* 464, 231–239. doi:10.1042/BJ20140606.

Ashkenazy, H., Abadi, S., Martz, E., Chay, O., Mayrose, I., Pupko, T., et al. (2016). ConSurf 2016: an improved methodology to estimate and visualize evolutionary conservation in macromolecules. *Nucleic Acids Res.* 44, W344–W350. doi:10.1093/nar/gkw408.

Bandekar, S. J., Arang, N., Tully, E. S., Tang, B. A., Barton, B. L., Li, S., et al. (2019). Structure of the C-terminal guanine nucleotide exchange factor module of Trio in an autoinhibited conformation reveals its oncogenic potential. *Sci. Signal.* 12, eaav2449. doi:10.1126/scisignal.aav2449.

Chen, Z., Guo, L., Sprang, S. R., and Sternweis, P. C. (2011). Modulation of a GEF switch: Autoinhibition of the intrinsic guanine nucleotide exchange activity of p115-RhoGEF. *Protein Sci.* 20, 107–117. doi:10.1002/pro.542.

Derewenda, U., Oleksy, A., Stevenson, A. S., Korczynska, J., Dauter, Z., Somlyo, A. P., et al. (2004). The Crystal Structure of RhoA in Complex with the DH/PH Fragment of PDZRhoGEF, an Activator of the Ca2+ Sensitization Pathway in Smooth Muscle. *Structure* 12, 1955–1965. doi:10.1016/j.str.2004.09.003.

He, X., Kuo, Y.-C., Rosche, T. J., and Zhang, X. (2013). Structural Basis for Autoinhibition of the Guanine Nucleotide Exchange Factor FARP2. *Structure* 21, 355–364. doi:10.1016/j.str.2013.01.001.

Kristelly, R., Gao, G., and Tesmer, J. J. G. (2004). Structural Determinants of RhoA Binding and Nucleotide Exchange in Leukemia-associated Rho Guanine-Nucleotide Exchange Factor. *J. Biol. Chem.* 279, 47352–47362. doi:10.1074/jbc.M406056200.

Lavery, R., and Sacquin-Mora, S. (2007). Protein mechanics: a route from structure to function. *J. Biosci.* 32, 891–898. doi:10.1007/s12038-007-0089-x.

Lucato, C. M., Halls, M. L., Ooms, L. M., Liu, H., Mitchell, C. A., Whisstock, J. C., et al. (2015). The Phosphatidylinositol (3,4,5)-Trisphosphate-dependent Rac Exchanger 1·Ras-related C3 Botulinum Toxin Substrate 1 (P-Rex1·Rac1) Complex Reveals the Basis of Rac1 Activation in Breast Cancer Cells. *J. Biol. Chem.* 290, 20827–20840. doi:10.1074/jbc.M115.660456.

Lutz, S., Shankaranarayanan, A., Coco, C., Ridilla, M., Nance, M. R., Vettel, C., et al. (2007). Structure of G q-p63RhoGEF-RhoA Complex Reveals a Pathway for the Activation of RhoA by GPCRs. *Science (80-.).* 318, 1923–1927. doi:10.1126/science.1147554.

Mitin, N., Betts, L., Yohe, M. E., Der, C. J., Sondek, J., and Rossman, K. L. (2007). Release of autoinhibition of ASEF by APC leads to CDC42 activation and tumor suppression. *Nat. Struct. Mol. Biol.* 14, 814–823. doi:10.1038/nsmb1290.

Murayama, K., Kato-Murayama, M., Akasaka, R., Terada, T., Yokoyama, S., and Shirouzu, M. (2012).

Structure of the Rho-specific guanine nucleotide-exchange factor Xpln. *Acta Crystallogr. Sect. F Struct. Biol. Cryst. Commun.* 68, 1455–1459. doi:10.1107/S1744309112045265.

Navizet, I., Cailliez, F., and Lavery, R. (2004). Probing Protein Mechanics: Residue-Level Properties and Their Use in Defining Domains. *Biophys. J.* 87, 1426–1435. doi:10.1529/biophysj.104.042085.

Petit, A.-P., Garcia-Petit, C., Bueren-Calabuig, J. A., Vuillard, L. M., Ferry, G., and Boutin, J. A. (2018). A structural study of the complex between neuroepithelial cell transforming gene 1 (Net1) and RhoA reveals a potential anticancer drug hot spot. *J. Biol. Chem.* 293, 9064–9077. doi:10.1074/jbc.RA117.001123.

Sacquin-Mora, S., Laforet, É., and Lavery, R. (2007). Locating the active sites of enzymes using mechanical properties. *Proteins Struct. Funct. Bioinforma.* 67, 350–359. doi:10.1002/prot.21353.

Shen, Y., Nedyalkova, L., Tong, Y., Tempel, W., Crombet, L., Arrowsmith, C. H., et al. Crystal structure of the DH and PH-1 domains of human FGD5.

Skowronek, K. R., Guo, F., Zheng, Y., and Nassar, N. (2004). The C-terminal Basic Tail of RhoG Assists the Guanine Nucleotide Exchange Factor Trio in Binding to Phospholipids. *J. Biol. Chem.* 279, 37895–37907. doi:10.1074/jbc.M312677200.

Snyder, J. T., Worthylake, D. K., Rossman, K. L., Betts, L., Pruitt, W. M., Siderovski, D. P., et al. (2002). Structural basis for the selective activation of Rho GTPases by Dbl exchange factors. *Nat. Struct. Biol.* 9, 468–475. doi:10.1038/nsb796.

Worthylake, D. K., Rossman, K. L., and Sondek, J. (2000). Crystal structure of Rac1 in complex with the guanine nucleotide exchange region of Tiam1. *Nature* 408, 682–688. doi:10.1038/35047014.

Xiang, S., Kim, E. Y., Connelly, J. J., Nassar, N., Kirsch, J., Winking, J., et al. (2006). The Crystal Structure of Cdc42 in Complex with Collybistin II, a Gephyrin-interacting Guanine Nucleotide Exchange Factor. *J. Mol. Biol.* 359, 35–46. doi:10.1016/j.jmb.2006.03.019.
